# Comparison of genomic and proteomic data in recurrent airway obstruction affected horses using ingenuity pathway analysis^®^

**DOI:** 10.1186/1746-6148-7-48

**Published:** 2011-08-15

**Authors:** Julien Racine, Vinzenz Gerber, Marybeth Miskovic Feutz, C Paige Riley, Jiri Adamec, June E Swinburne, Laurent L Couetil

**Affiliations:** 1 Department of Clinical Veterinary Medicine, Vetsuisse-Faculty, University of Berne, Länggassstrasse 124, Berne, 3012, Switzerland; 2 Department of Veterinary Clinical Sciences, School of Veterinary Medicine, Purdue University, 625 Harrison Street, West Lafayette, Indiana, 47907, USA; 3 Bindley Bioscience Center, Purdue University, 201 South University Street, West Lafayette, Indiana, 47907, USA; 4 Animal Health Trust, Lanwades Hall, Kentford, Newmarket, Suffolk CB8 7UU, UK

## Abstract

**Background:**

Recurrent airway obstruction (RAO) is a severe chronic respiratory disease affecting horses worldwide, though mostly in the Northern hemisphere. Environmental as well as genetic factors strongly influence the course and prognosis of the disease. Research has been focused on characterization of immunologic factors contributing to inflammatory responses, on genetic linkage analysis, and, more recently, on proteomic analysis of airway secretions from affected horses. The goal of this study was to investigate the interactions between eight candidate genes previously identified in a genetic linkage study and proteins expressed in bronchoalveolar lavage fluid (BALF) collected from healthy and RAO-affected horses. The analysis was carried out with Ingenuity Pathway Analysis^® ^bioinformatics software.

**Results:**

The gene with the greatest number of indirect interactions with the set of proteins identified is *Interleukin 4 Receptor (IL-4R)*, whose protein has also been detected in BALF. *Interleukin 21 receptor *and *chemokine (C-C motif) ligand 24 *also showed a large number of interactions with the group of detected proteins. Protein products of other genes like that of *SOCS5*, revealed direct interactions with the IL-4R protein. The interacting proteins NOD2, RPS6KA5 and FOXP3 found in several pathways are reported regulators of the NFκB pathway.

**Conclusions:**

The pathways generated with *IL-4R *highlight possible important intracellular signaling cascades implicating, for instance, NFκB. Furthermore, the proposed interaction between SOCS5 and IL-4R could explain how different genes can lead to identical clinical RAO phenotypes, as observed in two Swiss Warmblood half sibling families because these proteins interact upstream of an important cascade where they may act as a functional unit.

## Background

Recurrent airway obstruction (RAO) is a respiratory disease characterized by periods of airway obstruction caused by hyperresponsiveness to inhaled organic molds and endotoxins [1,2]. Clinically, affected horses exhibit a chronic, spontaneous cough, nasal discharge, and increased respiratory efforts associated with an elevation in maximal transpulmonary pressure change compared to healthy horses or horses with inflammatory airway disease (IAD) [3]. Diagnosis is based on history, clinical signs, and diagnostic tests. Endoscopic evaluation of RAO-affected horses reveals excessive mucopurulent exudate in the tracheobronchial tree [4]. Cytological analysis of bronchoalveolar lavage fluid (BALF) is characterized by non-septic inflammation with increase in mucus and neutrophils (> 25% of the total nucleated cell count) [2]. Various pulmonary function tests allow quantification of the degree of airway obstruction [3].

The immunological basis for RAO is controversial. A number of studies found that cytokine profiles are consistent with T_H_2 type response (e.g. interleukin (IL)-4, IL-13) [5-9]. Other studies, however, suggest that a T_H_1 response and cytokines (e.g. IL-8, IL-17) are responsible for neutrophil recruitment in RAO [10-16]. A study performed with horses affected by summer pasture-associated obstructive pulmonary disease (SPAOPD) revealed that the expression of T_H_1 and T_H_2 cytokines varies throughout the year [17]. The type and amount of key cytokines and other intracellular regulatory and transcription factors that are expressed upon contact with an antigen modulate the inflammatory response. Characterization of key interactions and pathways would be helpful in understanding the inflammatory response in RAO horses and whether it fits the rodent derived T_H_1/T_H_2 paradigm.

Several studies suggest a strong genetic basis with a complex mode of inheritance for RAO. Segregation and genomic analyses performed on two Swiss Warmblood families have led to the conclusion that the mode of inheritance of RAO is characterized by major gene effects, and that these genes differ between families. In the first of these families, RAO was transmitted in an autosomal recessive mode and the major association was found on equine chromosome 13 (ECA13), whereas in the second, it was transmitted in an autosomal dominant mode and the major association was found on ECA15 [18-20]. Interestingly, horses from both families showed no phenotypical differences in the expression of RAO, including clinical scores, endoscopic mucus scores, BALF and tracheo-bronchial secretion cytology, response to methacholine challenge and values of arterial oxygenation [21]. These results suggest genetic heterogeneity for the clinical phenotype RAO.

Proteomic and peptidomic analyses shed light on the metabolic status of biological systems and represent new approaches in the study of complex diseases like asthma and lung cancer in humans [22] and animal models of human diseases [23]. Recent research in proteomics improved disease phenotype characterization based on peripheral blood biomarkers or BALF cytokines in human suffering from asthma and chronic obstructive pulmonary disease [24,25]. One of the major challenges in proteomic analysis is the large amount of data generated, which makes bioinformatics software capable of processing the information indispensable [22].

For the present study, we used genomic and proteomic data previously collected from healthy and RAO-affected horses, and performed a comparison using bioinformatics software (Ingenuity Pathway Analysis [IPA^®^]). The tool "Path Explorer" was used to search for documented molecular interactions based on the Ingenuity^® ^Knowledge Base. This database contains millions of documented and published molecular interactions (Ingenuity^® ^Systems, http://www.ingenuity.com). Proteins present in BALF from RAO-affected horses and controls were identified by mass spectrometry [26] and these data were imported into IPA^®^. Information about eight candidate genes for RAO identified in a family-based whole-genome scan study [20] was also imported into IPA^® ^to identify documented pathways linking these candidate genes to the BALF proteins identified with proteomics. Thus, this study compares genomic and proteomic data within the framework of IPA^® ^in order to 1) identify the number of interactions between candidate genes for RAO and proteins detected by proteomic analyses and 2) characterize the interacting proteins and pathways involved.

## Results

The following candidate genes [20] were investigated for interactions with the set of proteins detected in BALF: *Interleukin 4 receptor (IL-4R), IL-21R, chemokine (C-C motif) ligand 24 (CCL24), IL-27, prostaglandin E receptor 4 (PTGER4), phosphodiesterase 4D (PDE4D), suppressor of cytokine signaling 5 (SOCS5) and IL-7R*.

IPA^® ^identified only a few direct interactions between the eight candidate genes and BALF proteins. Products of the following four gene candidates, *SOCS5, IL-7R, PTGER4, and PDE4D*, were predicted to directly interact with proteins identified in BALF. Direct interactions between protein products of candidate genes and detected proteins were the following: *SOCS5 *interacts with IL-4R, *IL-7R *with forkhead box P3 (FOXP3), and *PTGER4 *as well as *PDE4D *with arrestin beta 1 (ARRB1). ARRB1 and IL-4R are downregulated and FOXP3 is upregulated in RAO horses according to proteomic analysis. No other interactions were identified.

From a total of 277 proteins identified in the proteomics study, 56 (20.2%) were reported to have indirect interactions with *IL-4R*, 33 (11.9%) with *IL-21R*, 18 (6.5%) with *CCL24*, and 3 (1.1%) with *IL-27*.

**Figure 1:**
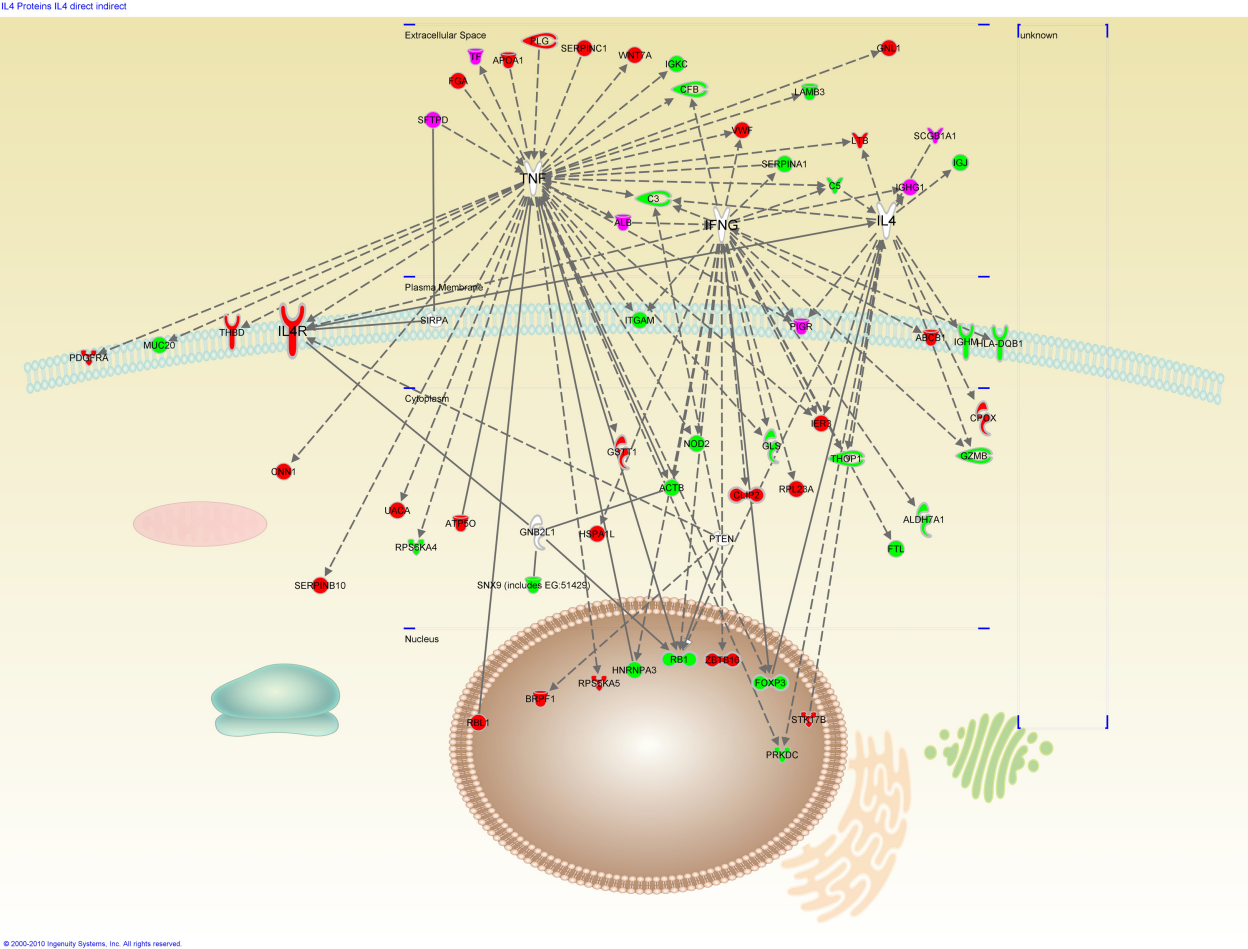
**interactions between IL-4R and proteins**. Red: downregulated in RAO, green: upregulated in RAO, magenta: controversial results, arrow: acts on, line: binds to, continuous line: direct interaction, dotted line: indirect interaction.

To identify the maximum number of possible interactions affiliated with IL-4R, IPA^® ^proposed 6 "connecting proteins" based on known pathways: tumor necrosis factor (TNF), interferon gamma (IFNG), interleukin 4 (IL-4), guanine nucleotide binding protein (G protein), beta polypeptide 2-like 1 (GNB2L1), signal-regulatory protein alpha (SIRPA), and phosphatase and tensin homolog (PTEN) (Table 1). According to proteomics results, 26 (46.4%) of the 56 interacting proteins are downregulated in RAO horses, while 24 (42.9%) are upregulated. Six proteins (10.7%) show controversial expression from the proteomics results (some peptides up- as well as downregulated in RAO). Twenty (35.7%) interacting proteins are located in the extracellular space, 8 (14.3%) in the cell membrane, 19 (33.9%) in the cytoplasm, and 9 (16.1%) in the nucleus (Figure 1). Extracellular proteins are involved in lungs' innate immunity (SERPINA1; SFTPD, TF, CFB, C3, C5) and in acquired immunity (IGHG1, IGJ, IGKC), amongst other functions (Table 2). One extracellular protein in particular, lymphotoxin beta (LTB), also operates as a member of the Tumor Necrosis Factor family. The majority of cell-membrane proteins participate in immune processes such as adhesive interactions of granulocytes (ITGAM), while others are immunoglobulins (IGHM) or proteins of the Major Histocompatibility Complex class II (HLA-DQB1). Such heterogeneity of functions can also be seen in cytoplasmic proteins. These proteins are involved in the induction of NF-κB (NOD2), protection of cells from oxidative stress (ALDH7A1, GSTT1), or participate in cell lysis and in cell-mediated immune responses (GZMB). Proteins present in the nucleus, on the other hand, are assumed to play a role in the regulatory mechanisms of apoptosis, cancerogenesis (RBL1, RB1), immune-regulation (FOXP3) and regulation of NF-κB activity (Table 2). Figure 1 illustrates the different types of interactions between IL-4R and other proteins.

**Table 1:** Indirect interactions between genes *IL-4R, IL-21R, CCL24, IL-27 *and proteins

Gene, (abbreviation) and [number of interactions]	Indirect Interactions	Connecting proteins
***interleukin 4 receptor*** ***(IL-4R)*** **[56]**	**Downregulated in RAO**: FGA; APOA1; PLG; SERPINC1; WNT7A; GNL1; VWF; LTB; PDGFRA; THBD; GSTT1; ABCB1; STK17B; CPOX; CLIP2; CNN1; RPL23A; BRPF1; RBL1; UACA; RPS6KA5; HSPA1L; IER3; ATP50; ZBTB16; SERPINB10; **Upregulated in RAO**: IGKC; CFB; LAMB3; C3; SERPINA1; C5; MUC20; ITGAM; IGHM; HLADQB1; RPS6KA4; SNX9; ACTB; NOD2; GLS; THOP1; FTL; ALD7H7A1; GZMB; IGJ; HNRNPA3; RB1; FOXP3; PRKDC; **Controversial findings**: TF; SFTPD; SCGB1A1; ALB; IGHG1; PIGR;	SIRPA; TNF; IFNG; IL-4; GNB2L1; PTEN
***interleukin 21 receptor*** ***(IL-21R)*** **[33]**	**Downregulated in RAO**:VWF; IL-4R; IER3; ZBTB16; APOA1; CLIP2;RPL23A; RBL1; FGA; HSPA1L; SERPINC1; ATP50, ABCB1; **Upregulated in RAO**: C5;SERPINA1; CFB; ITGAM; HNRNPA3; RB1; ACTB; NOD2; FOXP3; ALDH7A1; FTL; GLS; GZMB; THOP1; HLA-DQB1; ITGAM **Controversial findings **:ALB; SFTPD; PIGR;	IFNG; TNF; CD40LG
***chemokine (C-C motif) ligand 24*** ***(CCL24)*** **[18]**	**Downregulated in RAO**:STK17B; IER3; IL-4-R; LTB; CPOX; **Upregulated in RAO**: C5; IGJ; C3; IGHM; GZMB; THOP1; RB1; PRKDC; FOXP3; **Controversial findings **: PIGR; SCGB1A1; IGHG1;	IL-4
***interleukin 27*** ***(IL-27)*** **[3]**	**Upregulated in RAO**: HLA-DQB1; C5; FOXP3	

**Table 2:** Interaction summary of IL-4R with proteins (source of protein functions: Uniprot: http://www.uniprot.org

Compartment	Function	Proteins
**Extracellular**	Lung's innate immunity	SERPINA1; surfactant protein D (SFTPD), transferrin (TF), complement factor B (CFB), complement component 3 (C3), complement component 5 (C5)
	
	Immunoglobulins	immunoglobulin heavy constant gamma 1 (IGHG1), immunoglobulin J polypeptide, linker protein for immunoglobulin alpha and mu polypeptides (IGJ); immunoglobulin kappa constant (IGKC)
	
	Tumor Necrosis Factor family	lymphotoxin beta (TNF superfamily, member 3) (LTB)
	
	Other proteins	apolipoprotein A-I (APOA1), wingless-type MMTV integration site family member 7A (WNT7A), laminin beta 3 (LAMB3), Albumin (ALB) and secretoglobin, family 1A, member 1 (SCGB1A1) fibrinogen alpha chain (FGA); Plasminogen (PLG); serpin peptidase inhibitor clade C (antithrombin) member 1 (SERPINC1); von Willebrand factor (VWF); serpin peptidase inhibitor clade A (alpha-1 antiproteinase antitrypsin), member 1 (SERPINA1)

**Cell membrane**	Immunoglobulin	immunoglobulin heavy constant mu (IGHM)
	
	Immunoglobulins-receptor	polymeric immunoglobulin receptor (PIGR)
	
	MHC class II	major histocompatibility complex, class II, DQ beta 1 (HLA-DQB1)
	
	Adhesive interactions (e.g. granulocytes)	integrin, alpha M (complement component 3 receptor 3 subunit) [ITGAM]
	
	Hemostasis	thrombomodulin (THBD)
	
	Other proteins	ATP-binding cassette, sub-family B (MDR/TAP), member 1 (ABCB1) mucin 20, cell surface associated (MUC20); platelet-derived growth factor receptor, alpha polypeptide (PDGFRA)

**Cytoplasm**	Respiratory chain enzyme	ATP synthase, H+ transporting, mitochondrial F1 complex, O subunit (ATP5O);
	
	Induction of NF-kappa-B	nucleotide-binding oligomerization domain containing 2 (NOD2)
	
	Protection from oxidative stress	aldehyde dehydrogenase 7 family, member A1(ALDH7A1); glutathione S-transferase theta 1 (GSTT1)
	
	necessary for target cell lysis in cell-mediated immune responses	granzyme B (granzyme 2, cytotoxic T-lymphocyte-associated serine esterase 1) (GZMB)
	
	Other Proteins	calponin 1, basic, smooth muscle (CNN1); serpin peptidase inhibitor, clade B (ovalbumin), member 10 (SERPINB10); uveal autoantigen with coiled-coil domains and ankyrin repeats (UACA); ribosomal protein S6 kinase, 90 kDa, polypeptide 4 (RPS6KA4); sorting nexin 9 (SNX9); heat shock 70 kDa protein 1-like (HSPA1L); actin, beta (ACTB); Glutaminase (GLS); ribosomal protein L23a (RPL23A); immediate early response 3 (IER3); thimet oligopeptidase 1 (THOP1); ferritin, light polypeptide (FTL); coproporphyrinogen oxidase (CPOX)

**Nucleus**	Tumor suppressors	retinoblastoma-like 1 (p107) (RBL1); retinoblastoma 1 (RB1)
	
	Represses transcription of NF-kappa-B in response to TNF	ribosomal protein S6 kinase, 90 kDa, polypeptide 5 (RPS6KA5); forkhead box P3 (FOXP3)
	
	Probable transcription factor	forkhead box P3 (FOXP3)
	
	positive regulator of apoptosis	serine/threonine kinase 17b (STK17B)
	
	Involved in DNA repair mechanism	protein kinase, DNA-activated, catalytic polypeptide (PRKDC);
	
	Other Proteins	bromodomain and PHD finger containing, 1 (BRPF1); heterogeneous nuclear ribonucleoprotein A3 (HNRNPA3); zinc finger and BTB domain containing 16 (ZBTB16)

Apart from CD40 ligand (CD40LG), which is only present in the *IL-21R *pathway, "connecting proteins" proposed by IPA and indirectly interacting proteins present in these two pathways always appear in the *IL-4R *pathway. Protein product of *IL-27 *was found in three indirect interactions, namely HLA-DQB1, C5, and FOXP3 (Table 1).

## Discussion

The main conclusions drawn from this comparative analysis of genomic and proteomic data are as follows: 1) four of the eight candidate genes, namely *SOCS5, IL-7R, PTGER4*, and *PDE4D*, are implicated in direct interactions with proteins identified in BALF; 2) *IL-4R*, *IL-21R *and *CCL24 *are related to the identified BALF proteins by a large number of indirect interactions; 3) these indirect interactions highlight intracellular regulatory mechanisms that might play central roles in RAO pathophysiology (e.g. NF-κB); 4) the interaction of SOCS5 with IL-4R might explain how genomic heterogeneity in RAO-affected horses results in the same phenotype.

*SOCS5 *encodes for a protein which directly interacts with IL-4R. This gene is one of the main RAO-candidates in Swiss Warmblood family 2 [20] and is located in a region of ECA15 associated with the disease. SOCS5 downregulates the cytokine signal transduction by terminating intracellular signaling in a variety of ways. This can happen with the ubiquitination and subsequent degradation of the Janus kinase [JAK] and signal transducers and activators of transcription [STAT] receptors. SOCS5 is predominantly produced in T_H_1 cells and inhibits differentiation toward the T_H_2 cell type. In humans, its role has been implicated in the pathogenesis of allergic asthma [27,28]. Interestingly, the fact that SOCS5 and IL-4R directly interact might give a molecular explanation as to why the disease is clinically indistinguishable in both families [21]. Genetic variations in *IL4R *and *SOCS5 *may have various effects during RNA replication or splicing, or may influence protein function and stability. A molecular explanation for disease expression might be that a *SOCS5 *variant could lead to a protein with a different biological activity that could modulate IL-4R function and lead to the same effect as an *IL-4R *mutation. As IL-4R and SOCS5 are located upstream in the signaling cascades, genetic mutations of the coding genes might lead to the same phenotype, thus explaining genetic heterogeneity in RAO.

*PTGER4 *and *PDE4D *are located in a region of ECA21 associated with RAO in both Swiss Warmblood family 1 and 2. They directly interact with an important intracellular molecule that was detected during proteomic analysis, namely ARRB1. ARRB1 not only inhibits G protein-coupled receptor signaling, but also upregulates gene transcription of *B-cell CLL/lymphoma 2*. This anti-apoptotic factor enhances the survival of CD4+ T cells and might be one explanation for autoimmunity in humans suffering from multiple sclerosis [29]. Finally, *IL-7R*, a candidate gene from ECA21, directly interacts with FOXP3 which is a regulatory T-cell transcription factor implicated in the pathophysiology of human asthma.

*IL-4R *was examined in this study both as an RAO-gene candidate and as one of the proteins detected by proteomic evaluation of BALF. *IL-4R *was previously identified as an RAO candidate gene on ECA13 in Swiss Warmblood family 1 [19,20]. Also, *IL-4R *was found to have the greatest number of indirect interactions with the proteins detected in the BALF according to the pathways suggested by IPA^®^.

Another RAO-gene candidate from the same chromosomal region, *IL-21R*, shares homologies with the *IL-4R alpha chain*, and functions along a similar pathway. Since these two genes might be the most promising of all RAO-candidate genes, focusing on the *IL-4R*-pathway is likely to produce valuable insights into the signaling cascades ultimately leading to the disease. The three most crucial "connecting proteins" to this pathway are the TNF-family, IFN-γ, and IL-4, and thus deserve special attention.

The TNF-family consists of three members: TNF-α, lymphotoxin-α (LTA), and lymphotoxin-β (LTB). A large number of proteins identified by proteomic analysis of BALF (39) interact with the TNF-family in the IL-4R pathway and it is noteworthy that LTB was also identified in BALF. These cytokines are mostly membrane-bound, produced by macrophages and T cells, and fulfill multiple functions within immune response mechanisms. Mast cells can also produce large amounts of TNF-α [30] and interactions between these cells may be involved in neutrophil recruitment to the airway mucosa. Important functions of TNF-α that may play a role in the pathophysiology of RAO, are neutrophil recruitment and induction of proinflammatory cytokines via activation of the NFκB pathway. The proteins ITGAM, NOD2, RPS6KA5, and FOXP3 that were detected in the proteomic analysis substantiate the importance of the TNF-family and NFκB pathways. TNF-proteins interact with receptors of the tumor necrosis factor receptor family (TNFR), which in turn communicate with an intracellular signaling pathway known as Tumor Necrosis Factors Receptor Associated Factors (TRAFs). Upregulation of TNF-α leads to a rapid externalization of granules containing P-selectin (Weibel-Palade bodies), to an increased expression of E-selectin, and to a strong expression of the intercellular adhesion molecule 1 (ICAM-1) in endothelial cells. ITGAM is located on the surface of neutrophils and interacts with ICAM-1. These mechanisms are critical events in neutrophil extravasation. On an intracellular level, TNF-receptor activation leads to apoptosis or to signals that promote new gene expression through activation of NFκB. NOD2 is a cytoplasmic protein that recognizes bacterial proteoglycans and then activates NFκB. Both nuclear factors RPS6KA5 and FOXP3 repress transcription of NFκB. FOXP3 is a transcription factor expressed by regulatory T cells. These cells are able to modulate the activation of other T cells (i.e. T_H_1 and T_H_2) and play a key role in immune homeostasis [28]. In humans affected by asthma, FOXP3 protein expression within CD4+CD25high T-cells is significantly decreased compared to controls [31]. Cytokines like IL-8 and TNF-α are induced by NFκB activation [32,33] and may play a role in neutrophil recruitment during an RAO crisis. This is illustrated by a study that detected high levels of NFκB activity in bronchial cells of RAO-affected horses in comparison to healthy horses [34]. Therefore, our data highlight several factors that all deserve further investigations in order to evaluate their impact in RAO-pathophysiology, especially regarding the neutrophils recruitment, one of the key mechanisms leading to this disease.

The IPA^® ^platform generates pathways based on experimentally identified genes or proteins reported in the literature. In this study, for each pathway, 277 BALF proteins identified using proteomics and one candidate gene were specified so that IPA^® ^could indentify interactions. Furthermore, "connecting proteins" were added by the software in order to expand pathways. This approach presupposes that the likelihood of a candidate gene playing a pivotal role in the pathophysiology of RAO increases if that gene stands in multiple interactions with proteins detected in the proteomic analysis. The "connecting proteins" TNF, IFN-γ, and IL-4 act at the origin of signaling cascades and display pleiotropic effects. Hence their effective concentrations may be low or undetectable, which would explain why they were not detected in the proteomic analysis. Protein expression might also differ depending on whether tissue samples (i.e. biopsies) or BALF were examined. Furthermore, the heterogeneity and dynamic nature of RAO suggest that additional proteomic analysis of BALF from a wide range of naturally-occurring cases of RAO is warranted before results from this study may be generalized and the implicated proteins and their effects on the lower airways of horses can be confirmed. Another issue is that IPA^® ^builds connections based on literature from humans, mice, rats and cell cultures. Although fundamental immune and inflammatory pathways are conserved, there may be some important interspecies differences. Furthermore, transcriptomic and metabolomic analyses might provide much-needed information about smaller molecules (e.g. cytokines, metabolites) and their regulatory effects on the pathophysiology of RAO, and thus constitute another important research target. For instance, microarray assays and differential display polymerase chain reaction (DD PCR) have already been used to explore differential gene expression in RAO [35,36]. Ultimately, integration of information gained from all of these approaches may be needed to better understand the complex molecular pathogenesis of RAO.

## Conclusion

The integration of proteomic and genomic data on RAO, collected by two independent approaches, has produced a new set of data that identifies novel interactions which emphasize a central role for IL-4R and SOCS5 and implicate downstream intracellular signaling cascades. Indeed, these insights formulate the first molecular hypotheses on a proteomic level to explain the observation of genetic heterogeneity in RAO. This study thus illustrates the value of bioinformatics software for the analysis of large and complex sets of data and offers new approaches for the study of complex immunological diseases such as RAO.

## Methods

### Whole genome scan study

For the genetic studies, phenotypes were classified using HOARSI (Horse Owner Assessed Respiratory Signs Index), as described in detail elsewhere [37]. Briefly, horse owners were contacted by phone and informed consent obtained. Only horses with clinical signs that had persisted for at least 2 months were included in the study. All horses were 5 years or older with a history of hay feeding. A standardized questionnaire was used to gather information from the horse owners on the animals' history of chronic coughing, increased breathing effort at rest, and nasal discharge. This information was combined into a HOARSI 1-4. The classification refers to the period when the horses were exhibiting the most severe clinical signs. While HOARSI 1 comprises unaffected individuals (severity class 1) RAO in this study is represented in severity class 3, which comprises HOARSI 3 and 4 individuals [37]. Validation on 33 offsprings of sire 1 and 36 offsprings of sire 2 using comprehensive clinical examination showed that HOARSI 3 and 4 individuals in exacerbation are fully consistent with the RAO phenotype [21].

Horses selected for genetic linkage analyses arose from two Warmblood sire half sibling families exhibiting high RAO prevalence. The two Warmblood sires showed obvious clinical signs of respiratory distress (nostril flare, increased abdominal lift, or increased respiratory rate) and airway obstruction when stabled in stalls with straw bedding and fed hay, and showed remission of these signs when stabled in a barn complex especially adapted to the requirement of RAO patients (bedding of dust-free shavings; haylage feeding). A total of 248 horses were included in a whole-genome linkage analysis study using microsatellite markers. The study was approved by the animal use committee of the canton of Berne, Switzerland. These two families and the genetic analyses have been described in detail elsewhere [20,37]. Briefly, a total of 286 microsatellite markers covering the 31 horse autosomes and ECAX were used for this study. Fragment amplification using PCR was followed by fragment-length measurement on an ABI 3100 (Applied Biosystems), and genotypes were called using GeneMapper ver. 4.0 (Applied Biosystems). The genotyping data were analyzed using QTL Express [38], GRID QTL (http://www.gridqtl.org.uk/)[39] and FASTLINK [20,40].

### Proteomics study

#### Horses

Five horses previously diagnosed with Recurrent Airway Obstruction (RAO) and six age-matched healthy horses with no history of respiratory disease were used in this study. A diagnosis of RAO was made based on maximum change in transpulmonary pressure (ΔP_Lmax_) > 15 cmH_2_O, and > 25% neutrophils in BALF cytology during disease exacerbation. Horses also had reversible airway obstruction documented by pulmonary function tests following bronchodilator administration or environmental change [2]. The control horses had no clinical signs attributable to chronic respiratory disease when housed indoors and fed hay and no history of infectious respiratory disease (fever, nasal discharge, and cough) in the past 3 months. All procedures were approved by the Purdue University Animal Care and Use Committee.

All horses were maintained on pasture for at least two months with no dry hay supplementation before the beginning of this study. On Day 1, horses were transported from the pasture to the laboratory and allowed at least 30 minutes to acclimate to the lab environment. The evaluation consisted of a complete physical examination and calculation of a clinical score, standard pulmonary function testing (PFT), and BALF cytology. After the horses recovered from sedation, each pair (one RAO and one control) were stalled in adjacent stalls for the exposure trial.

In order to induce signs of acute airway obstruction in the RAO-affected horses, all horses were fed moldy hay and pelleted feed and were bedded on straw. The moldy hay was shaken in the breathing zone of each horse for two minutes twice a day until the clinical score of the RAO-affected horse reached 10 (out of 21 possible; Tesarowski et al. 1996). When the RAO-affected horse had a clinical score of ≥ 10, PFT were performed as previously described [41]. When the RAO-affected horse had a ΔP_Lmax _> 15 cmH_2_O, the tests performed on Day 1 were repeated on the RAO-affected horse and its age-matched control. Upon completion of the second set of tests, all horses were returned to pasture with no access to dry hay. Collection of BALF was performed after all lung function measurements were obtained [3].

#### Proteomics sample preparation

The BALF supernatant was filtered through sterile gauze and stored at -80°C in 1 mL aliquots until further analysis. Protein concentration in the BALF supernatant was measured with a BCA Assay (Thermo Scientific Pierce BCA Protein Assay Kit, Thermo Fisher Scientific, Inc., Rockford, IL, USA). For each sample, 100 μg of protein was incubated with three volumes of cold acetone at -20°C for 30 minutes to precipitate proteins. The samples were centrifuged for two minutes to concentrate the protein pellet, and the supernatant was discarded. The samples were lyophilized to complete dryness (approximately 15 minutes). Denaturation solution (8 M urea + 10 mM Dithiothreitol, 10 μL) was added to each sample and incubated for 90 minutes at 37°C. Ammonium bicarbonate (2 μL, 100 mM) and reducing cocktail (10 μL of a solution of 195 μL acetonitrile, 1 μL triethylphosphine, and 4 μL 2-iodoethanol) were added to each sample and incubated at 37°C for 90 minutes. The samples were lyophilized overnight. The following day, each sample was resuspended in 80 μL of 100 mM ammonium bicarbonate. Trypsin was added at a ratio of 1 gram trypsin to 50 grams protein (2 μg of 0.5 μg/μL trypsin) to each sample, and incubated overnight at 37°C. On the final day, 1 μL of 10% trifluoroacetic acid (TFA) was added to each sample to stop the digestion. Each sample was run on a C18 column (C-18 Vydac, 300 A, The Nest Group, Southborough, MA, USA) to remove the majority of the salt from the sample, according to standard laboratory procedure. After the final column wash, the samples were lyophilized overnight. Samples were processed in batches, and the peptide pellets were stored at -80°C until all samples were processed. All samples were reconstituted in 100 μL of 0.01% TFA (final concentration 1 μg/μL) before mass spectrophometric analysis.

#### LC-MS Analysis

The peptides were separated on a nanoLC-Chip system (1100 Series LC equipped with HPLC Chip interface, Agilent, Santa Clara, CA, USA). After injection of 1 μg of sample, the peptides were concentrated in the on-chip 300SB-C18 enrichment column and washed with buffer A (5% acetonitrile, ACN/0.01% TFA) at flow rate of 4 μl/min for 5 minutes. The enrichment column was switched into the nano flow path and further separated with the on-chip C-18 reversed phase ZORBAX 300SB-C18 analytical column (0.075 μm × 43 mm; Agilent, Santa Clara, CA, USA) coupled to the electrospray ionization (ESI) source of the ion trap mass spectrometer (XCT Plus; Agilent, Santa Clara, CA, USA). The column was eluted with a 55 minute linear gradient from 5%-35% buffer B (100% acetonitrile, 0.01% TFA) at a rate of 600 nl/min, followed by a 10 minute gradient from 35%-100% buffer B. The column was re-equilibrated with an isocratic flow (5% buffer B) at 600 nl/min. ChemStation software was used to control the system (Agilent, Santa Clara, CA, USA). LC-MS chromatograms were acquired in positive ion mode under the following conditions: a capillary voltage of 1850 V and an end plate offset of 500 V. The dry temperature was set at 300°C. Dry gas flow was maintained at 4 L/min. Acquisition range was 350-2200 m/z with 0.15 second maximum accumulation time and scan speed of 8100 m/z per second.

#### Statistical Analysis

The raw data from the LC-MS were pre-processed before analysis to eliminate artifacts such as noise, peak broadening, instrument distortion, etc. [42]. Briefly, spectral deconvolution was performed to filter noise in the spectra and to separate overlapping peptide peaks. Peak alignment was used to adjust for retention time drift between samples over the time course of the LC-MS data acquisition. After the pre-processing was complete, the mean intensity of each peak was calculated for each group (RAO-affected and control) and the fold change of each peptide between groups was calculated. All statistical analysis was performed with the Purdue Discovery Pipeline (PDP) (Purdue University, West Lafayette, IN).

#### LC-MS/MS Analysis

MS/MS analysis was performed on one RAO-affected horse and one control horse. To identify differentially expressed peptides, automated MS/MS spectra were acquired during the run in the data-dependent acquisition mode with the selection of the three most abundant precursor ions (0.5 min active exclusion; 2+ ions preferred). The MS/MS files acquired on the ion trap mass spectrometer were uploaded to Spectrum Mill protein identification software (Agilent, Santa Clara, CA) and searches were performed using Spectrum Mill and the NCBI database. The parameters were as follows: no more than two tryptic miscleavages allowed, cysteine searched as ethanol cysteine, variable oxidized methionine, 2.5 Da peptide tolerance and 0.7 Da mass tolerance. Only peptides with a score of 5 or higher were considered true positives.

#### Peak identification

Peak identification was performed for one RAO-affected horse and one control horse. The output files from the PDP statistical analysis and Spectrum Mill peptide identification were merged into one file. The PDP mass to charge ratio (m/z) and retention time (RT) data (peaks) for one horse was matched with the Spectrum Mill identification data (peptide and protein names) for the same horse. First, peaks were matched with peptides within 3 m/z units and 3 minutes RT. Then, for peptides matched to multiple peaks, a single peak was selected based on charge (from SM data and manual review of spectra), closest m/z, and closest RT. For peaks matched to multiple peptides, a single peptide was selected based on the highest Spectrum Mill score and percent scored peak intensity (% SPI) and the lowest Spectrum Mill reverse score.

### Generation of pathways with IPA^®^

The functional analysis of a network identified the biological functions and/or diseases that were most significant to the molecules in the network. The network molecules associated with biological functions and/or diseases in Ingenuity's Knowledge Base were considered for the analysis. Right-tailed Fisher's exact test was used to calculate the probability that each biological function and/or disease assigned to that network is due to chance alone (Ingenuity^® ^Systems, http://www.ingenuity.com).

All proteins identified in proteomic analyses of BALF from RAO and control horses were included. The reason was that we first intended to explore their global interaction with the candidate genes (i.e. their protein products). A total of 8 candidate genes (*IL-4R, IL-21R, CCL24, IL-27, PTGER4, PDE4D, SOCS5, IL-7R*) from the genetic linkage analysis and 277 proteins identified in the proteomics studies of RAO horses were uploaded into the IPA software (Ingenuity^® ^Systems, http://www.ingenuity.com) to generate pathways. *IL-4R, IL-21R, CCL24, IL-27 *are located on ECA13 and are associated with disease in Swiss Warmblood family 1; *SOCS5 *is located in a region of ECA15 and is associated with disease in family 2; *IL-7R, PTGER4 *and *PDE4D *are on ECA21 and are associated with disease in both family 1 and 2, although less significantly [20]. Search results were restricted to molecular interactions described in lung tissues, immune cells, BALF and sputum, genetic disorders, hypersensitivity, immunological diseases, infectious diseases, and inflammatory diseases in humans, mice, rats and cell cultures. Possible types of interaction were "binds to", "inhibits", "acts on", "leads to" and "translocates to". Interactions can also be direct or indirect. Direct interactions were defined as two molecules making direct physical contact with each other with no intermediate step. Direct interactions also included chemical modifications such as phosphorylations, provided that there was evidence that the two factors involved interact directly rather than through an intermediate. Indirect interactions are genetic or molecular relationships explicitly reported in the literature and not inferred. For instance, up-regulation of TNF-α leads to a strong expression of the intercellular adhesion molecule 1 (ICAM-1) in endothelial cells without direct physical contact between TNF-α and ICAM-1.

## Authors' contributions

JR carried out the comparison of genomic and proteomic analyses using IPA and drafted the manuscript.

VG participated in the design of the study and contributed to genomic studies.

MMF collected BALF and carried out the proteomic analysis of BALF.

CPR contributed to the proteomic studies from BALF and statistical analysis of proteomics data.

JA participated in the design of the study and statistical analysis of proteomics data.

JES carried out the genomic studies.

LLC conceived the study and participated in its design and coordination and helped to draft the manuscript.

All authors read and approved the final manuscript.
